# Flavonoid and Organosulphur Phytoconstituents From Allium Sativum Inhibits Antiapoptotic Protein Bcl-2: A Computational Molecular Modeling Study

**DOI:** 10.1200/GO.22.31000

**Published:** 2022-05-05

**Authors:** Mutiat Ibrahim, Abayomi Adegboyega, Rotimi Adegboro, Margaret Ilomuanya, Fatimah Abdulkareem

**Affiliations:** ^1^ University of Lagos, Lagos, Nigeria; ^2^ University of Jos, Jos, Nigeria

## Abstract

Apoptosis resistance to anticancer treatments can occur due to over-expression of Bcl-2 anti-apoptotic proteins. Studies have shown the use of Allium sativum (garlic) as a herbal supplement among cancer patients. In this study, the inhibitory activity of phytochemicals from Allium sativum against Bcl-2 anti-apoptotic protein was demonstrated using a computational technique.

**METHODS:**

Identified phytoconstituents from A. sativum were docked in receptor grid generated active sites of Bcl-2 (PDB-ID: 4AQ3) protein using the Glide-Ligand docking tool of Schrödinger Maestro 12.5. Receptor-ligand complex pharmacophore models were generated using the PHASE module, and the binding free energy of the complex was calculated using the MM-GBSA Prime panel. Potential lead compounds were screened for drug likeness using the Lipinski rule of five (RO5) and Veber rule. Absorption, distribution, metabolism, excretion, and toxicity (ADME/T) predictions of the leads were carried out using the PROTOX-II tool. Induced fit docking simulation was performed on the top ranked hit compound.

**RESULTS:**

Extra precision docking results showed that myricetin, kaempferol, and apigenin (flavonoid); α and β-phellandrene (cyclic monoterpene); 3-vinyl-4H-1,2-dithiin, 3-vinyl-1,2-dithiin and 2-vinyl-4H-1,3-dithiine (organosulphur) were ranked highest, with docking scores range from -6.00 to -3.78 kcal/mol compared with -3.62 kcal/mol demonstrated by co-crystallized ligand. Apigenin (CID: 5280443), 3-vinyl-1,2-dithiin (CID: 10219489), and 3-vinyl-4H-1,2-dithiin (CID: 150636) were identified as the top three lead compounds with free binding energies of -33.83, -30.36, and -29.70 kcal/mol respectively. The identified lead compounds from A. sativum were in accordance with RO5 and Veber rule with good oral bio-availability and ADME/T profile. Similar to the co-crystallized ligand, apigenin interacted with important active site amino acid residues like Try 67, Asp 70, Leu 96, Arg 105, Ala 108 among others.

**CONCLUSION:**

Some bioactive phytochemicals from A. sativum can be explored for development toward inhibition of antiapoptotic protein Bcl-2 in apoptosis resistance to anticancer treatments.

